# Unusual differential cross sections for the H + D_2_O → D + HOD exchange reaction induced by the C_3V_ transition state and quantum interference†

**DOI:** 10.1039/d5sc03277f

**Published:** 2025-07-10

**Authors:** Shu Liu, Qun Chen, Kejie Shao, Bina Fu, Dong H. Zhang

**Affiliations:** a State Key Laboratory of Molecular Reaction Dynamics, Dalian Institute of Chemical Physics, Chinese Academy of Sciences Dalian Liaoning 116023 China liushu1985@dicp.ac.cn zhangdh@dicp.ac.cn; b University of Chinese Academy of Sciences Beijing 100049 China

## Abstract

Previous experimental and theoretical studies have shown that direct reactive systems typically exhibit backward-peaked differential cross sections (DCS) at relatively low collision energies, while complex-forming reactive systems tend to display forward-backward symmetric DCS. Is this a universal phenomenon in all direct reactions, especially those proceeding through non-collinear transition states? In this work, we developed the quantum wave packet method to calculate the full-dimensional state-to-state DCSs for the title exchange reaction with D_2_O in the ground rovibrational state on a highly accurate neural network potential energy surface. For the first time, we obtain a sideward-scattered angle distribution just above the threshold, which directly reflects the C_3V_ transition state geometry of this reaction. As the collision energy increases, the DCS broadens and undergoes a series of notable changes, culminating in the dominance of backward scattering at *E*_c_ = 1.4 eV, accompanied by an early-sideward scattering peak. Although trajectory analysis can explain most of the DCS variations, significant differences persist between the quantum and quasiclassical trajectory DCSs, arising from quantum interference between the contributions from low and high partial waves. Additionally, the collision energy dependent DCSs at the scattering angle of 107° exhibit two clear step-like features around *E*_c_ = 0.91 and 1.16 eV, which can be attributed to the shape resonance states trapped in the C_3V_ well. In the energy region considered here, the majority of the available energy goes into the translational motion of the products, and the reaction exhibits low vibrational mode-specific behavior.

## Abstract 2

For the first time, a sideward-scattered angular distribution is observed just above the threshold, directly reflecting the 107° bending angle in the C_3V_ transition state of the title reaction.

## Graphical abstract

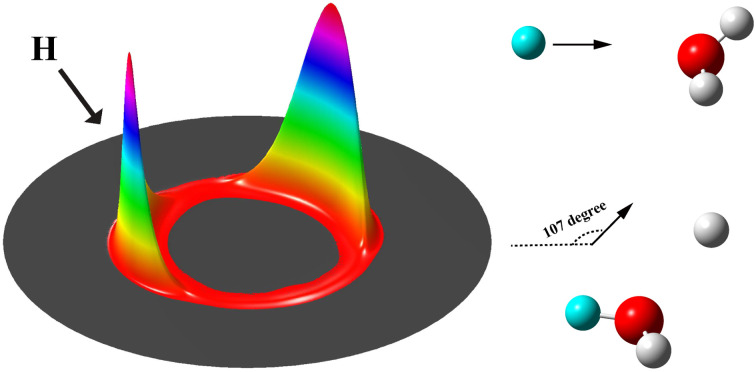

The state-to-state differential cross sections (DCSs) are reactant and product fully quantum state-resolved quantity, characterizing the scattering angle distribution of the reaction. They provide the most intuitive reflection of the reaction mechanism and are therefore the fundamental goal of both experimental and theoretical studies in chemical reaction dynamics. According to previous experimental and theoretical studies, the DCSs for a direct reactive system are backward peaked at relatively low collision energies, as observed in abstract reactions such as H + H_2_, H + H_2_O, and H + CH_4_,^1–3^ as well as the H + HCl and H + HBr exchange reactions.^4,5^ As the collision energy increases, the peak width broadens, or the peak gradually shifts to a smaller angle. The situation becomes complicated in hydrogen abstraction reactions involving the F atom, where the HF vibrational excited adiabatic potential wells in the post-barrier region support dynamical resonances that can affect the product scattering behavior.^6–13^ Moreover, for complex-forming reactive systems dominated by barrierless reaction pathways through deep wells, the DCS is typically forward–backward symmetric due to the long-lived reaction intermediates, such as those in the insertion reactions of O(^1^D), C(^1^D), and S(^1^D) with the ground state H_2_ molecule,^14,15^ and in the H + O_2_ and O(^1^D) + CO_2_ reactions.^16–18^ Nonstatistical effects have also been observed in many complex-forming reactions, stemming from the relatively short lifetime of the reaction intermediate.^14,16,19–24^

However, we wonder: are the DCSs of the direct reactions always backward peaked at low collision energies in all cases, especially in reactions with non-collinear transition states? The experiments and quasiclassical trajectory (QCT) studies of the oxygen isotope exchange reaction O(^3^P) + CO_2_ have provided some clues; however, the energies they studied were so high that they still led to backward scattering.^25^ The H + H_2_O exchange reaction and its isotopic analogue, which features a saddle point close to a C_3V_ geometry, present another opportunity for investigation.

The H + H_2_O reaction is the prototype for tetra-atomic reactions, in much the same way that the H + H_2_ reaction served as the prototype for triatomics. Theoretically, because three of the four atoms are hydrogens, the system is an ideal candidate for high quality *ab initio* calculations of a PES, as well as for accurate quantum reactive scattering calculations. Our group has calculated the fully converged DCS for the abstract channel H + H_2_O → H_2_ + OH and its isotopic analogue for both the initial ground and vibrational excited states.^2,26,27^ However, studies of the exchange channel have been limited to the total cross sections for an extended period. The integral cross sections (ICS) of the H + D_2_O → D + HOD reaction were first reported 25 years ago, in which the reaction probabilities were calculated with one OD bond in the D_2_O reactant treated as a spectator bond, and under the centrifugal sudden (CS) approximation for total angular momentum *J* > 0.^28^ It was later demonstrated that the CS approximation was inadequate for the H + H_2_O reaction.^29^ And although the spectator bond assumption works well for the H + H_2_O → H_2_ + OH abstraction reaction, both OH bonds should be treated as reactive bonds in order to accurately investigate the exchange process, due to the C_3V_ saddle point.^30^ In 2012, a full-dimensional quantum dynamics study for the H + D_2_O → D + HOD reaction was presented without any dynamical approximations. The exact coupled-channel (CC) ICSs agree well with the experimental results and show a distinct step-like feature just above the threshold, which provides the first evidence for a shape resonance in this reaction.^31^ In 2016, Zhao *et al.* performed a state-to-state quantum dynamics study for the H′ + H_2_O → H + H′OH reaction using the reactant-coordinate-based (RCB) method, but it was limited to total angular momentum *J* = 0.^32^

Here, we report the full-dimensional (6D) DCS for the H + D_2_O → D + HOD exchange reaction. To the best of our knowledge, this is the first quantum DCS for a polyatomic exchange reaction, although the state–state quantum dynamics calculations have been performed on many polyatomic reactions, with the development of both the initial state-selected^2,32–38^ and transition-state^39–41^ time-dependent wave packet (TDWP) methods. We observe a sideward-scattered angle distribution just above the threshold, which directly reflects the C_3V_ transition state geometry of this reaction. Moreover, the quantum interference effects are responsible for the significant difference between quantum mechanical (QM) and quasi-classical trajectory (QCT) DCS. Additionally, the shape resonances in this reaction lead to distinct step-like features in the collision-energy-dependent DCS at the scattering angle of 107°.

We utilized the multistep reactant-product-decoupling (MRPD) method^33,34^ to carry out the full-dimensional state-to-state quantum calculation on the CXZ potential energy surface (PES),^42^ which is the most accurate and smooth PES available for the OH_3_ reaction system, constructed by using the neural network (NN) method. To converge DCSs for collision energies up to 1.5 eV, we calculate state-to-state reaction probabilities for all total angular momenta *J* up to 20. For details of the numerical parameters, please refer to the ESI.† It should be noted that both OD bonds in the D_2_O reactant were treated as reactive in our calculations; that is, they were fully vibrationally excited in the interaction region. Moreover, the dividing surface used to extract the *S*-matrix elements is placed far enough in the product asymptotic region, where the two exchange channels are fully separated. Since the two D + HOD channels are equivalent, the reaction probabilities and cross sections presented in this study correspond to a single D exchange and should be doubled when compared to the experimental results.

Fig. 1(a) and (b) shows the total reaction probabilities for *J* = 0 and ICSs as a function of collision energy on the CXZ PES from the MRPD state-to-state calculations, compared with those obtained from the initial state selected wave packet (ISSWP) approach using only the reactant Jacobi coordinates. As can be seen, the agreement between these two results is excellent in the entire energy region considered, indicating the RPD partition of the wave function, as well as the continuous propagation, is highly accurate. The reaction probabilities exhibit a sharp peak just above the threshold, which remains as a clear step-like feature in the ICS, along with several broad peaks at higher energies that are nearly washed out in the ICS.

**Fig. 1:**
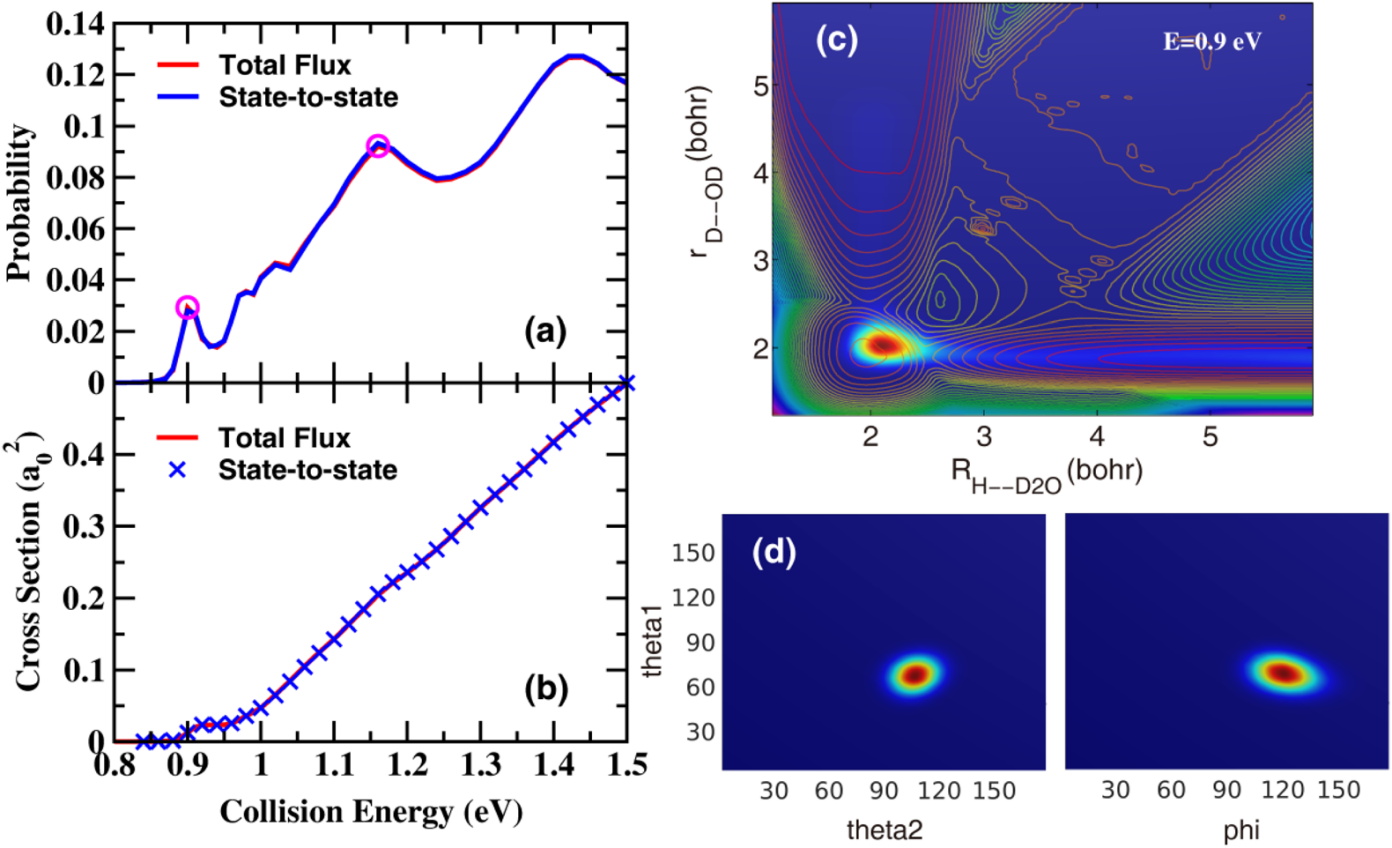
(a) Total reaction probabilities for *J* = 0 and (b) ICS for the title reaction on the CXZ PES obtained from the MRPD state-to-state calculations, in comparison with those obtained using the ISSWP approach. (c) Reactive scattering wave functions at the collision energy of 0.9 eV in the two Jacobi coordinates *R*_H-D_2_O_ and *r*_D-OD_ with other coordinates integrated. The contour lines are the corresponding 2D PESs along *R*_H-D_2_O_ and *r*_D-OD_ with other coordinates optimized. (d) Reactive scattering wave in the angle coordinates with *R*, *r*_1_, and *r*_2_ fixed at the peak position.

Fig. 1(c) displays the two-dimensional contour of the *J* = 0 scattering wave functions at the collision energy of 0.9 eV—the position of the sharp peak—obtained by Fourier transformation of the time-dependent (TD) wave functions after 4000 a.u. of propagation time. It can be seen that the scattering wave functions are localized in the C_3V_ well along the reaction coordinate. Examination of the wave function in the bending and torsion degrees of freedom, shown in Fig. 1(d), reveals no nodal structure. Furthermore, the Argand diagram for the ground state product, presented in Fig. S2,† shows a continuous counterclockwise motion without any kink. So the peak at 0.9 eV arises from a shape resonance, corresponding to a quasibound state trapped in the C_3V_ well—formed by the topography of the PES—on the ground-state vibrationally adiabatic potential (VAP), which decays into products by tunneling through the barrier. Fig. S3† shows the scattering wave functions at the collision energy of 1.16 eV, which are also localized in the C_3V_ well, with clear nodes along the *θ*_1_ and *φ* coordinates. So the broad peaks at higher energies originate from shape resonance states supported by the C_3V_ well on bending/torsion excited VAP.

Fig. 2 shows the DCSs at six collision energies, in terms of surface plots for the product translational energy and angle distributions for the reaction. The scattering angle is defined as the angle between the direction of the incoming H atom and the outgoing direction of the HOD product molecule. Unlike other direct reactions previously studied, the most intriguing observation in the title reaction is the sideward scattered DCS at the lowest energy of 0.88 eV, which peaked at 109° with a half-maximum width of 45°. As the collision energy increases, the DCS broadens, undergoing a series of remarkable changes and eventually becoming dominated by backward scattering at *E*_c_ = 1.4 eV, accompanied by an early-sideward scattering peak. Additionally, Fig. 2 provides an overview of the product vibrational state distributions. As shown, almost all HOD products populate in the (000) state below *E*_c_ = 1.0 eV. At *E*_c_ = 1.12 eV, the population of the (010) state increases considerably, although the (000) state still dominates the distribution. At *E*_c_ = 1.4 eV, the vibrational distribution broadens further. In addition to some populations in the first OD stretching (001) and (020) states, a few populations are also observed in states with excitation energy corresponding to the first OH stretching (100), as well as the (011) and (030) levels. The vibrational energy levels of the HOD, along with the collision energies at which they become populated, are summarized in Table S1.†

**Fig. 2:**
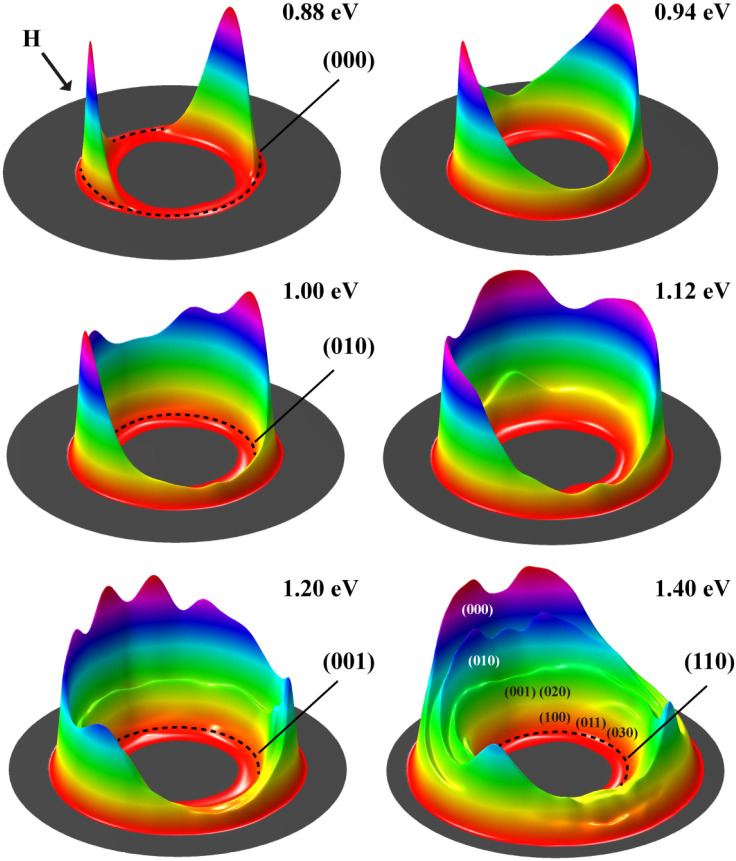
Surface plots for the product translational energy and angle distributions for the title reaction at the collision energy of 0.88, 0.94, 1.00, 1.12, 1.20, and 1.40 eV. The forward scattering direction for HOD corresponds to the direction of the incoming H atom. Dashed circles represent the maximum translational energies of the products in the indicated HOD vibrational states, which are labeled by (*n*_1_, *n*_2_, *n*_3_), where *n*_1_ is the quantum number for the OH stretching mode, *n*_2_ is for the bending mode, and *n*_3_ is for the OD stretching mode.

To quantitatively examine the variation of angle distribution with collision energy, we present the total DCSs, summed over all HOD rovibrational states, at a series of energies in Fig. 3. From 0.88 to 1.0 eV, the angular distribution broadens, with the peak shifting to a smaller angle. Meanwhile, the backward scattering components increase substantially, and the forward components also rise slightly. Subsequently, part of sideward scattering shifts toward the forward direction, while another part moves toward the backward direction, resulting in a flattening of the sideward peak at *E*_c_ = 1.12 eV. Between 1.16 and 1.2 eV, the sideward scattering component near 95° remains unchanged, while the forward- and backward-shifted components continue to grow, resulting in a concave shape in the sideward DCS. At collision energy above 1.2 eV, the backward scattered products, arising from two distinct sources, gradually become dominant. The peak position of early-sideward scattering shifts to 59° at *E*_c_ = 1.4 eV.

**Fig. 3:**
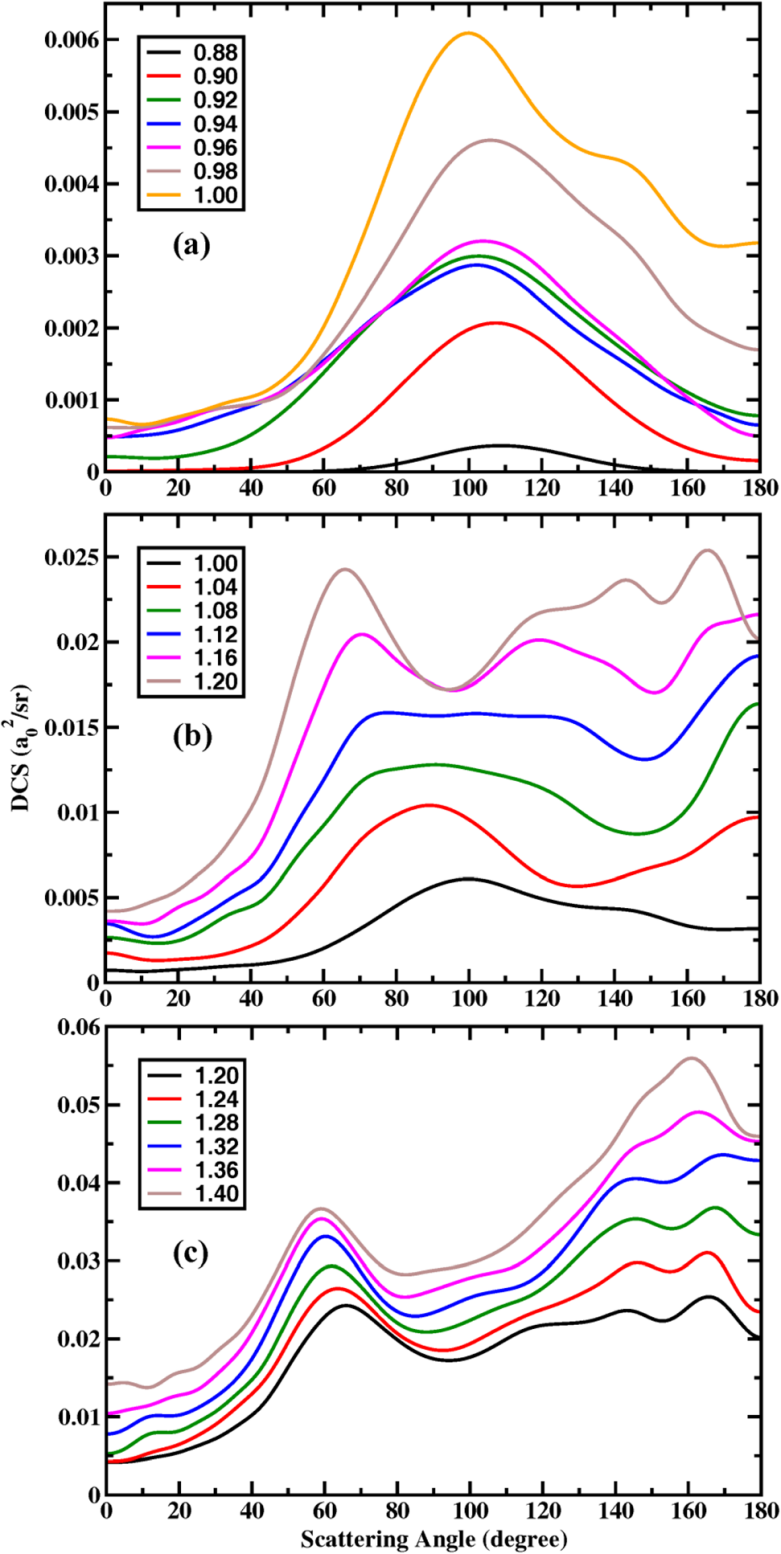
Total DCSs, summed over all HOD rovibrational states, for collision energies from 0.88 to 1.0 eV (a); from 1.0 to 1.2 eV (b); and from 1.2 to 1.4 eV (c).

To analyze the reaction mechanism, we also performed the QCT calculations on the CXZ PES. The details of QCT methodology are described in the ESI.†Fig. 4 compares the QM and QCT total DCSs at the collision energies of 0.90, 1.00, 1.20, and 1.40 eV. At 0.9 eV, the two scattering angle distributions are quite similar, both peaking near 107°. As the collision energy increases, the QCT DCS also broaden and develop pronounced backward scattering components. However, the QM and QCT scattering angle distributions show significant differences at higher collision energies. It can be seen that sideward scattering remains dominant in the QCT DCS at 1.2 and 1.4 eV, although the peak position shifts to 130° at 1.4 eV. Notably, the QCT DCSs do not reproduce the oscillatory structures observed in the QM results.

**Fig. 4:**
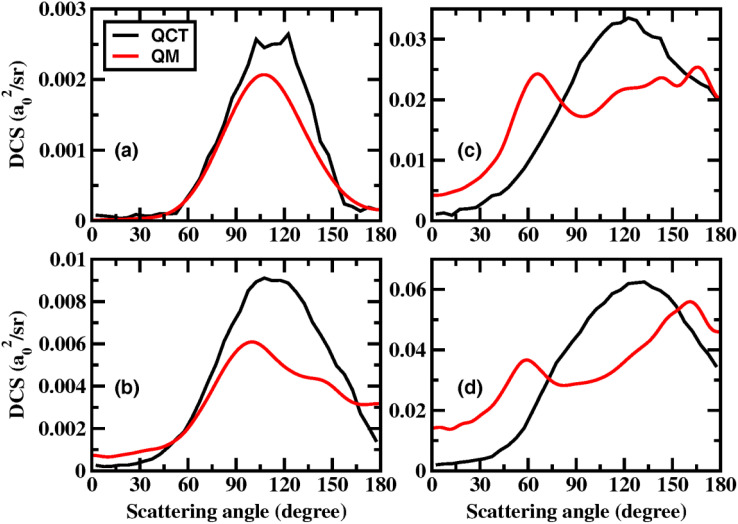
Total DCSs at the collision energy of (a) 0.90, (b) 1.00, (c) 1.20, and (d) 1.40 eV obtained by both QM and QCT calculations.

Fig. 5(a) and (b) show the QCT and QM deflection functions, which reveal the correlation between the total angular momentum *J* (or the impact parameter) and the scattering angle, for all product rovibrational states at *E*_c_ = 1.2 eV. To better correspond with the trajectories, the functions are presented without multiplication by sin *θ*. The QM generalized deflection function (GDF), which is additive over *J* and contains coherences between different values of *J*, was proposed by Prof. Jambrinato and Prof. Aoiz as^43^
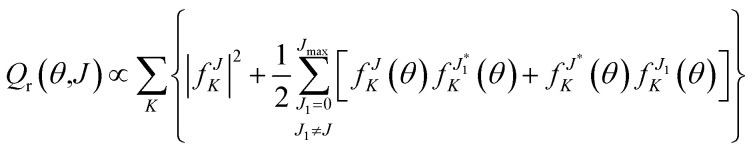
*K* is the projection of *J* on the product body-fixed *z* axis. Unlike the typical band-shaped profile of a direct reaction (where low *J* corresponds to backward scattering and high *J* to forward scattering), the classical deflection function for the title reaction shows a distinctive heart-shaped pattern. The initial sideward scattering peak is correlated with low *J*, while the backward and early-sideward scattering components are correlated with high *J*. The QM GDFs closely resemble their classical counterparts, indicating that most variations in the QM DCS can be accounted for by the trajectories.

**Fig. 5:**
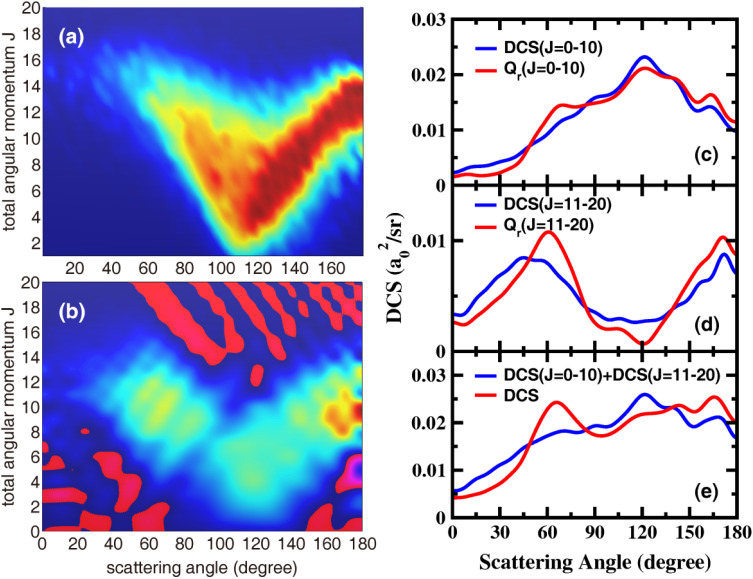
The QCT (a) and QM (b) deflection functions (not weighted by sin *θ*) for all the product rovibrational states at the collision energy of 1.20 eV. (c–e) The comparisons between the partially summed QM DCSs and partial QM deflection functions.

Analysis of the reactive trajectories at *E*_c_ = 1.2 eV revealed the reaction mechanisms during the collision process. (The snapshots and animations of the typical trajectories for each case are depicted in Fig. 6 and ESI Text.†) First, nearly all the reactive trajectories are direct, with the OH_3_ intermediate existing for less than one vibrational period. With small total angular momentum *J*, the direct exchange trajectories behave like nearly head-on collision with the shortest lifetime, where the angle between the directions of outgoing D atom and incoming H atom is close to the bending angle of 107° in the C_3V_ transition state. So the sideward-scattered HOD we obtained just above the threshold is a direct reflection of the transition state geometry in this exchange reaction. In contrast, the direct trajectories with large total angular momentum *J* lead to more glancing collision. In some of these glancing collisions, the incident H direction forms an angle with the OH bond of the transition state, causing the HOD to rotate and the sideways-scattered DCS to broaden in both the backward and forward directions. In the remaining glancing collision, the product D atom departs along the direction of the incoming H regent, which lies in the *D*_3h_ plane of the transition state, producing a backward-scattered HOD product.

**Fig. 6:**
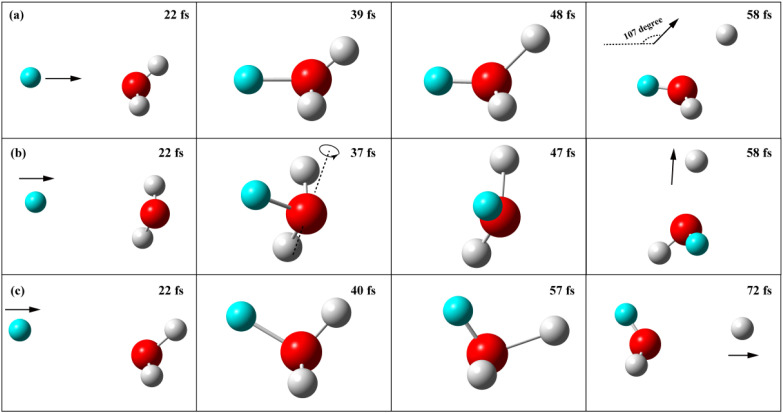
Snapshots of trajectories at *E*_c_ = 1.2 eV resulting in the sideward scattering with a small initial impact parameter of 0.24 bohr (*J* = 3) (a); the backward scattering with large impact parameters of 1.194 bohr (*J* = 15) (b) and 1.206 bohr (*J* = 15) (c). Each panel shows the geometry at the reaction time indicated (fs).

Despite their overall similarity to the QCT deflection functions, the QM GDFs also display noticeable oscillations and negative values (shown in red) across all scattering angles as shown in Fig. 5(b). These features are associated with constructive and destructive interference and are likely responsible for the oscillatory structures observed in the QM DCS. Fig. 5(c) and (d) depict the partially summed QM DCS, DCS([*J*_*i*_,*J*_*k*_]), representing the angular distribution calculated for a selected subset of partial waves, in comparison with the partial QM GDF*Q*_r_(*θ*,[*J*_*i*_,*J*_*k*_]), obtained by summing *Q*_r_(*θ*,*J*) over the same *J* range. While the partially summed DCS includes only the coherences among partial waves within the selected range, the partial QM GDF also includes coherences outside this range.^43^ Consistent with the reaction mechanism analyzed by trajectories, the low-*J* range (*J* = 0–10) primarily contributes to sideways scattering, whereas the high-*J* range (*J* = 11–20) is associated with backward and early-sideward scattering. However, for both these *J* ranges, *Q*_r_ is smaller than the partial DCS in the sideways region but larger in the forward and backward regions, reflecting interference cancellation and interference growth, respectively. Fig. 5(e) presents the incoherent sum of DCS(*J* = 0–10) and DCS(*J* = 11–20), which is similar to the QCT DCS at 1.2 eV, featuring a sideward scattering peak around 122°. Therefore, the peaks and dips in the total QM DCSs, which are absent in the corresponding QCT ones, are the results of quantum interference between contributions from low and high partial waves.

In addition to quantum interference, we also want to study the influence of shape resonance on the DCS of the reaction. In our previous study,^10,35^ the collision-energy-dependent DCS in the backward scattering direction were successfully used to detect Feshbach resonances and heavy–light–heavy reactivity oscillations, as they are dominated by a small number of low *J* and can largely retain the oscillatory structures in the *J* = 0 reaction probabilities. For the title reaction, because the HOD product is first sideward scattered just above the threshold, we present the collision-energy-dependent DCS at the scattering angle of 107° in Fig. 7(a). As seen, the total DCS exhibits two clear step-like features around *E*_c_ = 0.91 and 1.16 eV, consistent with two prominent resonance peaks in the *J* = 0 reaction probability in Fig. 1(a), which are attributed to the shape resonance states supported by the shallow C_3V_ well along the reaction path. As shown in Fig. 3, the step-like feature around *E*_c_ = 0.91 eV, which is characterized by minimal changes in DCS with increasing collision energy, can be observed across all scattering angles. However, the step-like feature around *E*_c_ = 1.16 eV can only be found in a small range of sideward angles.

**Fig. 7:**
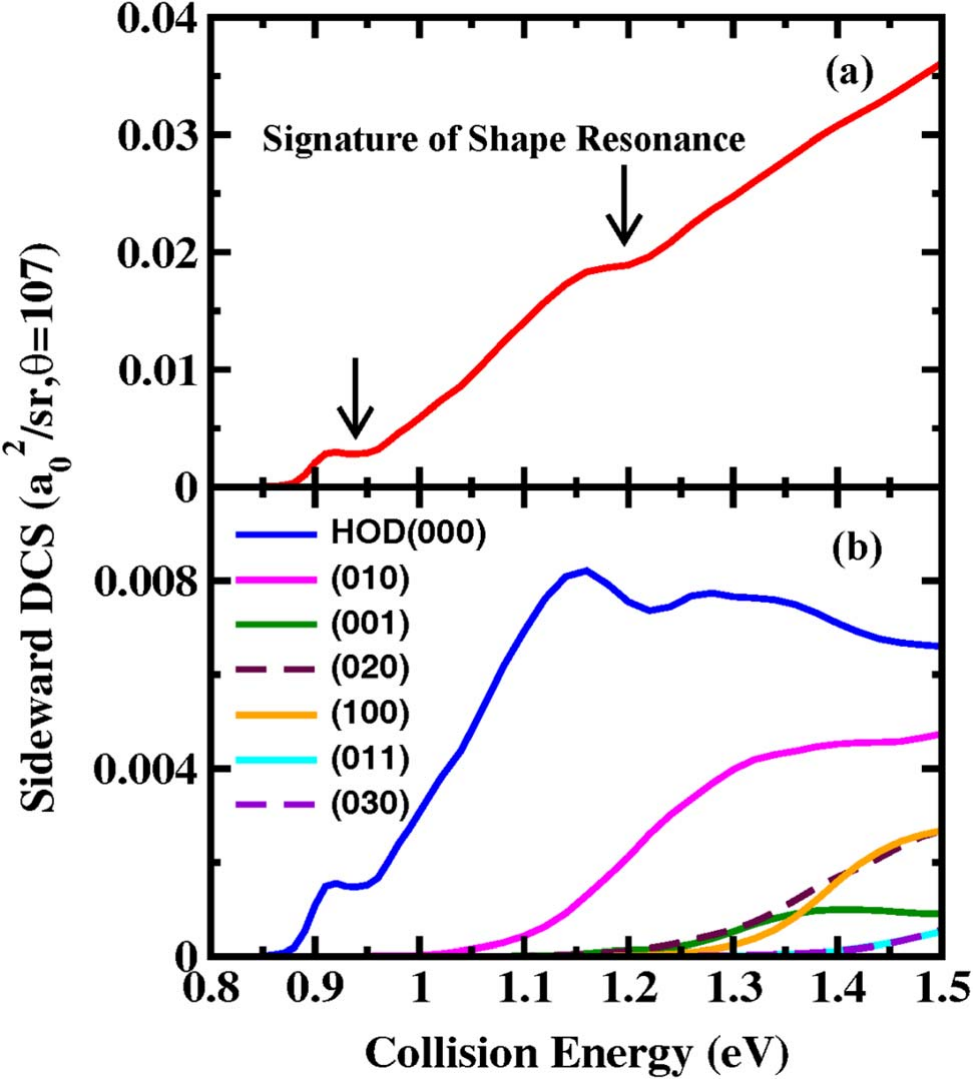
Collision energy dependent (a) total DCS and (b) product HOD vibrational state-resolved DCS at the scattering angle of 107°.

Fig. 7(b) shows the product vibrational state-resolved DCSs at the scattering angle of 107°. As can be seen, only the first seven vibrational states are populated in the energy region considered here. The majority of HOD is in the ground state, and the step-like features observed in the total DCS are primarily due to the ground state. As the collision energy increases, the contributions from vibrationally excited states increase. For HOD, because the OD stretching vibrational frequency is nearly double the bending frequency, there are strong Fermi resonances between levels (001) and (020), and their corresponding overtones. It is impossible to clearly distinguish between the strongly mixed states, therefore it is more appropriate to combine the populations of (001) and (020) states, as well as the (011) and (030) states, when comparing with experimental results. Our calculations predict that the ratio of population is 39.7 : 28.7 : 18.6(= 4.3 + 14.3) : 6.9 : 4.1(= 1.8 + 2.3) of (000) : (010) : [(001) + (020)] : (100) : [(011) + (030)] at *E*_c_ = 1.5 eV. Therefore, the reaction exhibits a low mode-specific behavior in the energy region considered here, with no clear preference for particular HOD bending or stretching modes. HOD is predicted to be distributed according to the sequence of the vibrational energy levels. This result clearly reflects the fact that the HOD angle and the newly formed OH bond in the C_3V_ transition state are only slightly smaller and slightly longer, respectively, than those in the HOD product.

Fig. 8 shows the fraction of the total available energy in the product channel that goes into the internal motion of HOD as a function of collision energy. As observed, the majority of the total available energy is directed into the translational motion of the products. The fraction of energy deposited into the vibrational motion first changes slowly, then increases considerably from 3% at *E*_c_ = 1.08 eV to 17.5% at *E*_c_ = 1.5 eV. Assuming that the HOD rotational constant remains relatively unchanged across different vibrational states, we can get the approximate fraction of rotational motion, which slightly increases and stays below 7% in the entire energy region.

**Fig. 8:**
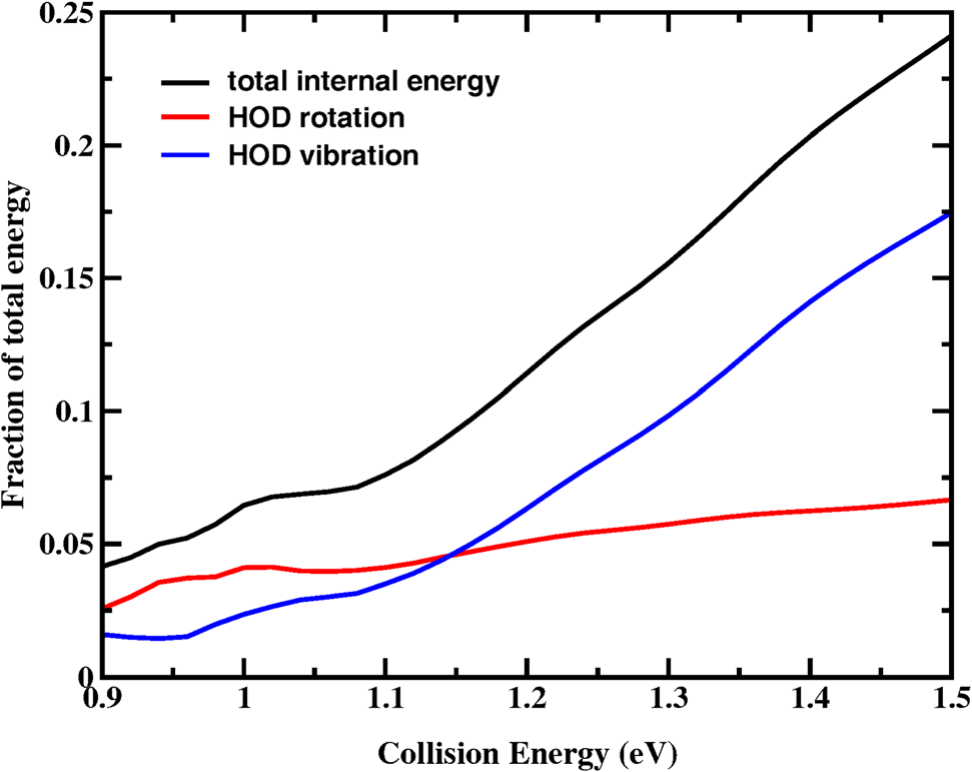
The fraction of the total available energy in the product channel going into the internal, rotation and vibration excitations of HOD as a function of the collision energy.

Therefore, our full-dimensional state-to-state quantum dynamics study reveals that the DCS of direct reactive systems are not always backward-peaked at relatively low collision energies. In the title exchange reaction, it directly reflects the non-collinear transition state geometry. We anticipate that this phenomenon exists in other non-collinear reactions and requires accurate theoretical and experimental investigations near the threshold. Moreover, the variations of the DCS with collision energy clearly reveal quantum effects in the reaction, including both interferences and shape resonances.

## Author contributions

S. L. and D. H. Z. conceived the research and wrote the manuscript. S. L. performed the quantum dynamical calculation. Q. C., K. S. and B. F. performed the QCT calculation.

## Conflicts of interest

There are no conflicts to declare.

## Supplementary Material

SC-OLF-D5SC03277F-s001

SC-OLF-D5SC03277F-s002

SC-OLF-D5SC03277F-s003

SC-OLF-D5SC03277F-s004

## Data Availability

All data are available in the main text, in the ESI†, or upon requests to the corresponding author.
