# Supplementary material for: Unusual differential cross sections for the H + D2O → D + HOD exchange reaction induced by the C3V transition state and quantum interference
Source: Chem Sci. 2025 Jul 10;16(33):14931–9. doi: 10.1039/d5sc03277f (PMC12282538; doi:10.1039/d5sc03277f)
Supplement: SC-016-D5SC03277F-s004 [file SC-016-D5SC03277F-s004.pdf]

Supplementary Information (SI) for Chemical Science.
This journal is © The Royal Society of Chemistry 2025

Supplementary Materials

Unusual differential cross sections for the H + D_2O → D + HOD exchange reaction induced by the C_{3V} transition state and quantum interference

Shu Liu[1,2]*#, Qun Chen[1,2#], Kejie Shao[1] and Dong H. Zhang[1,2]*

1) State Key Laboratory of Molecular Reaction Dynamics, Dalian Institute of Chemical Physics, Chinese Academy of Sciences, Dalian, Liaoning 116023, China

2) University of Chinese Academy of Sciences, Beijing 100049, China

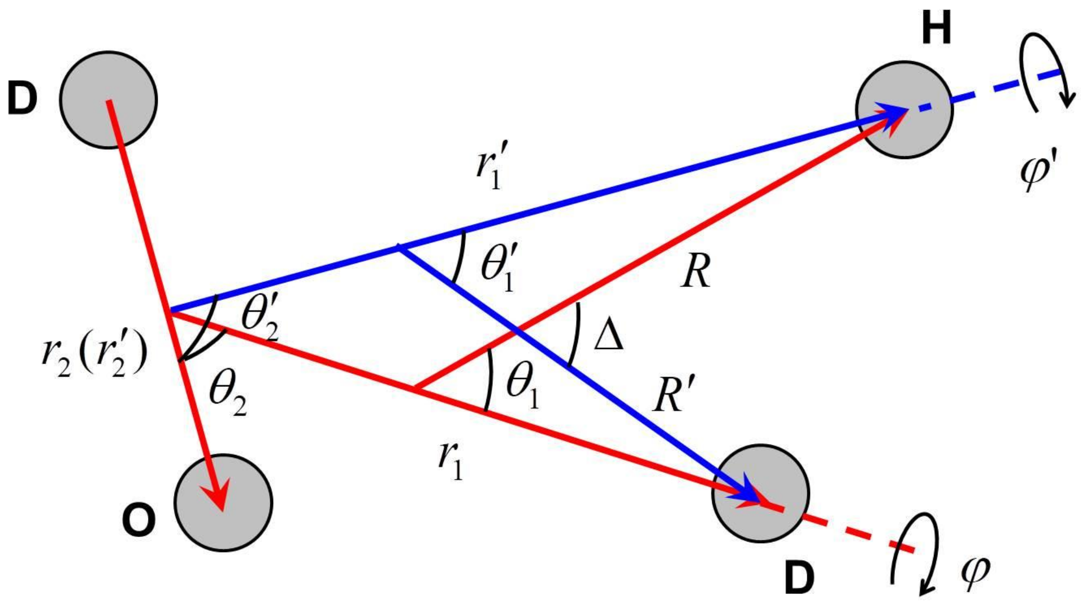

Fig. S1 Reagent Jacobi coordinates (R, r_1, r_2, θ_1, θ_2, φ) for the H + D_2O atom−triatom arrangement and product Jacobi coordinates (R', r_1', r_2', θ_1', θ_2', φ') for the D + HOD atom−triatom arrangement.

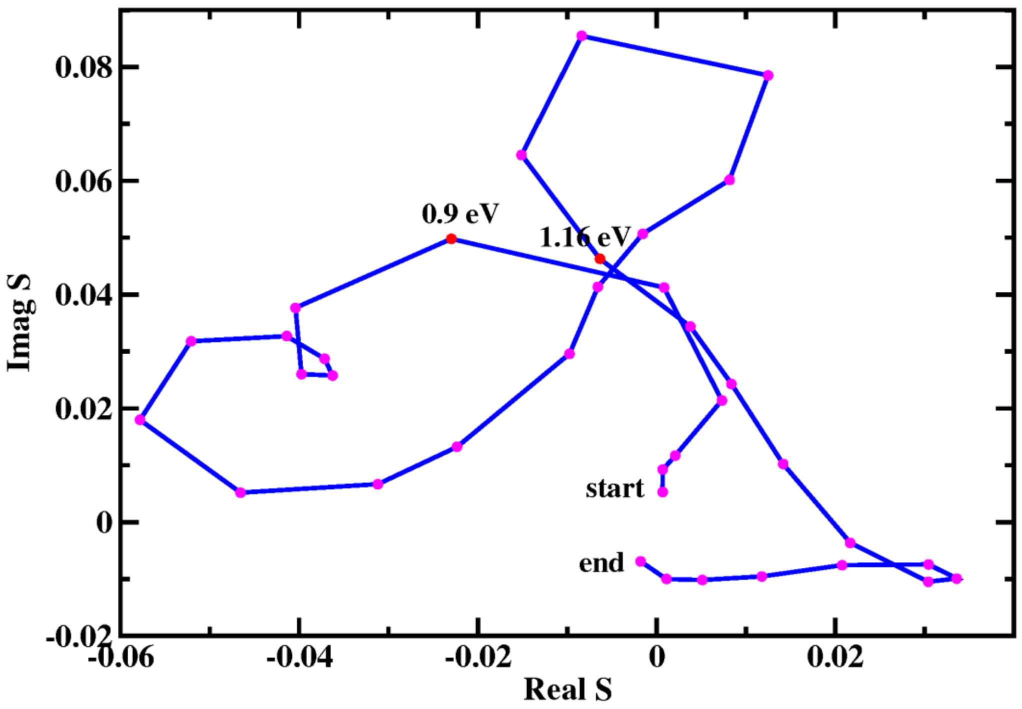

Fig S2 Argand diagram for the ground state product with total angular momentum J=0, over the collision energy range from 0.85 to 1.4 eV.

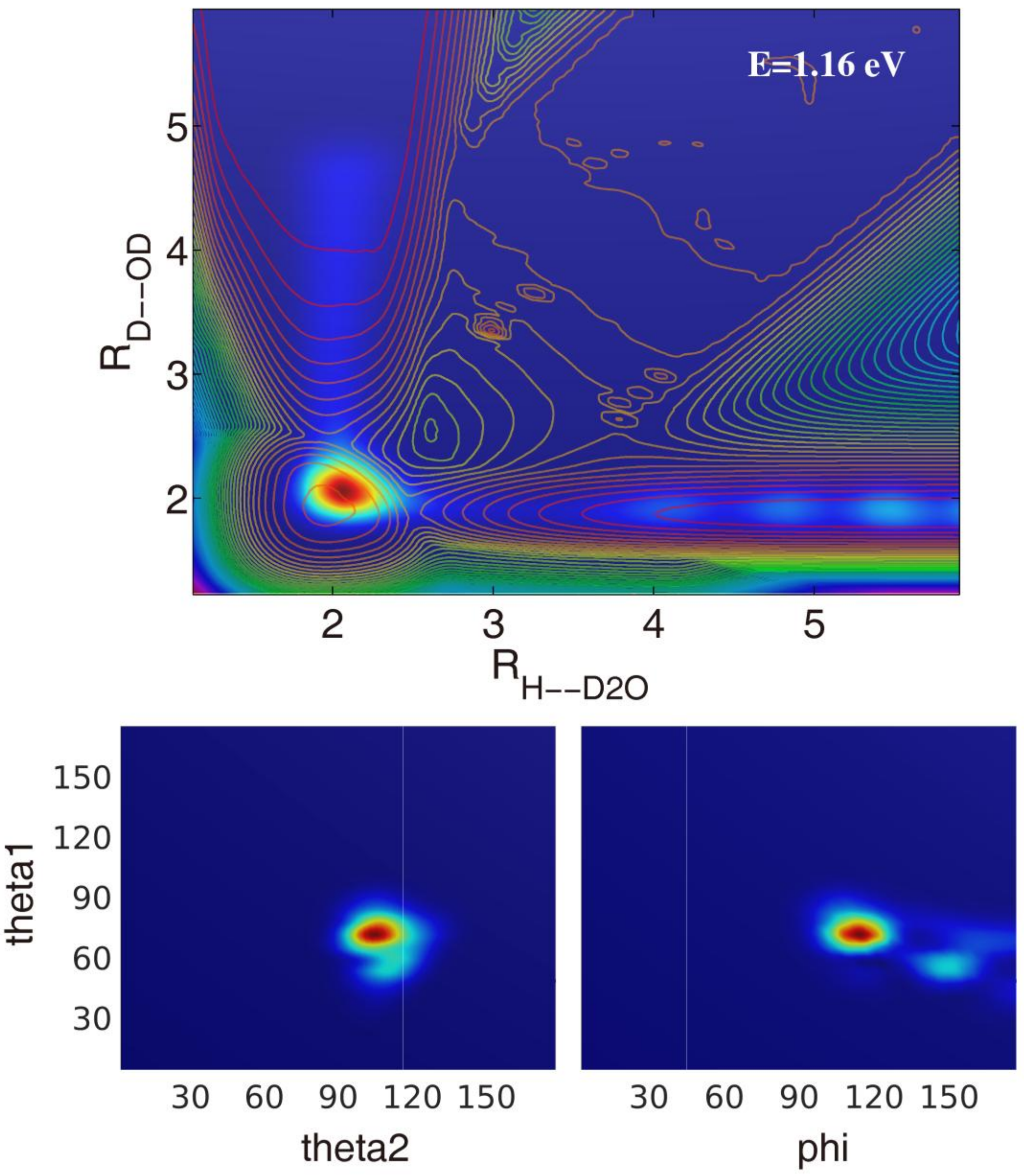

Fig S3 Reactive scattering wave functions at the collision energy of 1.16 eV in the two Jacobi coordinates $R_{H\text{-}D2O}$ and $r_{D\text{-}OD}$ with other coordinates integrated, and in the angle coordinates with R, r_1, and r_2 fixed at the peak position.

Table S1. The energy levels of the HOD vibrational states (with respect to the ground rovibrational state of HOD) and the collision energies at which they become populated.

HOD vibrational state	E_v (eV)	E_c (eV)
(000)	0	0.88
(010)	0.174	0.96
(001)	0.338	1.14
(020)	0.345	1.14
(100)	0.46	1.26
(011)	0.509	1.34
(030)	0.514	1.34

Animation From QCT Calculations

To illustrate the reaction mechanism, three animations of typical H + D_2O direct exchange trajectories at E_c=1.2 eV from quasiclassical trajectory (QCT) calculations are presented.

Animation S1: A direct exchange trajectory with small initial impact parameter leading to sideward scattering.

Animation S2: A direct exchange trajectory with large impact parameter leading to late-sideward scattering.

Animation S3: A direct exchange trajectory with large impact parameter leading to backward scattering.

Numerical parameters of quantum wave packet calculations

Figure S1 illustrates the reagent Jacobi coordinates (R, r_1, r_2, θ_1, θ_2, φ) for the H + D_2O arrangement, as well as the product Jacobi coordinates (R', r_1', r_2', θ_1', θ_2', φ') for the D + HOD arrangements. The numerical parameters used in the wave packet

propagation in reactant atom-triatom coordinates are as follows, with both OD bonds in the D_2O reactant treated as reactive bonds. The interaction region was defined by a rectangular box of [1.0,6.0]a_0 in the r_1 coordinate, [1.0,5.0]a_0 in the r_2 coordinate, and [1.0,6.0]a_0 for the R coordinate. The number of vibrational basis functions used was 65 for r_1 and 32 for r_2 coordinates. For the R coordinate, we used 40 sine discrete variable representation (DVR) points. The asymptotic region was defined from 6.0 to 12.0 a_0 with 42 sine DVR points for the R coordinate, and 8 vibrational basis functions for both the r_1 and r_2 coordinates. For the rotational motion, we used j_{1max}=28 and j_{2max}=50, which results in 18125 rotational basis functions for K=0 and even parity. The number of K blocks used in the calculation increases with the total angular momentum, from 1 for J=0, up to 8 for J=20. Thus, the maximum number of the rotational basis functions reaches 257714 in the 8 K-block case. The initial wave packet located at R_0=10 a_0. For lower J, we propagate the wave packets for 10000 a.u. of time with a time step of 10 to converge the reaction probability in the low energy region, especially near the resonance peak.

In product atom-triatom coordinates, we used a total number of 100 sine functions for the translational coordinate R' in a range of [3.0,15.0]a_0, 5 vibrational basis functions for r_1', and 3 vibrational basis functions for r_2'. We used j_{1max}'=28 and j_{2max}'=30, resulting in a total of 9425 rotational basis functions for K'=0 with even parity. The number of K blocks used for product propagation must be full (J+1) to get the accurate state-to-state probabilities. A dividing surface is placed at R'=10 a_0 to extract S matrix elements. To minimize the computational cost, the coordinate transformation, which transfers the absorbed wave packet in the reactant coordinates to the product coordinates, is carried out at every 8 propagation time steps.

The quasi-classical trajectory study

The quasi-classical trajectories (QCT) simulations were performed at collision energies ranging from 0.80 to 1.5 eV, with the D_2O reactant prepared in its ground rovibrational state. For each collision energy, the maximum impact parameter was

determined using 50,000 test trajectories. The initial H and D_2O were separated by 8.0 Å and the trajectories were terminated when products (or reactants for non-reactive trajectories) reached a separation of 8.0 Å. The propagation time step was 2 a.u., and the scattering parameters (vibrational phases and spatial orientation of initial reactants) were selected with a Monte Carlo approach. The gradient of the PES was obtained numerically by the central-difference algorithm. A total of 10,000,000 trajectories were simulated at each collision energy.
